# Trends in the Use of Medicare Home Health Care among Congregate Living Residents

**DOI:** 10.1016/j.jamda.2025.105498

**Published:** 2025-03-05

**Authors:** Jun Li, Bo Zheng, Brian McGarry

**Affiliations:** a Department of Public Administration and International Affairs, The Maxwell School of Citizenship and Public Affairs, Syracuse University, Syracuse, NY, USA; b Division of Geriatrics and Aging, Department of Medicine, University of Rochester Medical Center, Rochester, NY, USA

**Keywords:** Medicare, home health care, post-acute care, assisted living, congregate living

## Abstract

**Objective:**

To examine trends in home health care (HHC) use in congregate living and compare characteristics of HHC use between Medicare patients in congregate living and those in other home settings.

**Design:**

Retrospective cohort study describing HHC use, in and outside of congregate living, using national Medicare claims, assessment, and administrative data (2014–2019). We compared HHC use by setting, on HHC quality, planned visit quantity, referral source (post-acute or community-initiated), and recertifications. We additionally examined whether HHC patterns across settings differed by patient dual eligibility and race-ethnicity.

**Setting and Participants:**

Traditional Medicare (TM) and Medicare Advantage (MA) HHC patients aged 67 and older in congregate living or elsewhere.

**Methods:**

Multivariable regressions.

**Results:**

15.9% of HHC episodes in 2018–2019 were in congregate living, which increased 20.5% between 2014 and 2019. TM patients in congregate living were 4.4% (2.3 ppt, 95% CI: 1.7, 2.9) more likely to use high-quality agencies, had 8.7% (0.7 visits, 95% CI: 0.7, 0.8) more planned visits, were 27.6% (14.9 ppt, 95% CI: 14.7, 15.1) more likely to have community-initiated referrals, and 9.9% (3.2 ppt, 95% CI: 2.9, 3.5) more likely to be recertified compared with TM patients in other settings; differences were similar between settings among MA enrollees. Compared with their counterparts, dually eligible and racial-ethnic minoritized populations in congregate living were 2.6% (−1.5 ppt, 95% CI:−2.2,−0.8%) and 1.6% less likely (−0.9 ppt, 95% CI: −1.6, −0.3%) to use high-quality agencies, respectively, and dually eligible patients in congregate living had 6% fewer planned visits (−0.64 visits, 95% CI: −0.72, −55); these differences persisted across settings.

**Conclusion and Implications:**

Congregate living may have facilitated access to higher quality and quantity of HHC, but inequitably. Further research is needed to determine the value of high-frequency community-initiated HHC referrals in congregate living.

By 2030, 1 in 5 Americans will be older adults, with strong preferences to age at home.^1,2^ Two rapidly expanding community-based care sectors, skilled home health care [hereafter, home health care (HHC)] and congregate living, serve 3 million and 1 million older adults each year, respectively.^3,4^ As of 2014, around 10% of HHC patients lived in congregate settings.^5^ These patients receive intermittent skilled services (eg, nursing, therapy) and limited personal care through home health,^6^ whereas congregate living providers offer custodial care, medication management, health service coordination, and general supervision.^7^ A limited descriptive literature suggests that using both services might improve patient outcomes.^5^

How these services interact to support older adults remains largely unexplored. It is plausible that combining HHC and congregate living could lower provider costs or enhance care quality without increasing expenses. For instance, HHC may reduce the direct care burden for congregate living providers, especially for sicker or more socially disadvantaged individuals who might require more care. Conversely, congregate living providers could ease the workload for HHC agencies and reduce liability concerns that may stem from caring for patients who are otherwise at home alone or with limited family care. Additionally, congregate living providers could influence demand for specific agencies by helping patients identify high-quality agencies. In turn, agencies might prioritize congregate living patients to maintain referral relationships,^8^ potentially offering more visits or recertifications to extend caredespecially to disadvantaged populations who are less likely to access care in the community setting.^9,10^ Finally, agencies might also prefer congregate living patients because of the potential efficiency of providing visits to multiple patients living in the same location.

The incentives described in the prior paragraph also raise concerns about potential overuse of HHC, especially among traditional Medicare (TM) enrollees, who make up the majority of HHC patients.^11^ TM enrollees face no cost sharing for HHC and can receive unlimited episodes provided that a clinician certifies the need for skilled care and the patient is homebound. Medicare then pays agencies by the episode, which encourages more episodes per patient. With few deterrents to overuse, the Medicare Payment and Advisory Commission (MedPAC) has warned that some home health episodes could be wasteful, particularly those not following a hospitalization (ie, community-initiated episodes).^8,12^ In congregate living settings, where HHC benefits both congregate living providers and agencies, the potential for overuse may be particularly relevant.

Our objectives were therefore to (1) estimate the prevalence of HHC episodes in congregate living among Medicare enrollees [TM and Medicare Advantage (MA)] from 2014 to 2019, and (2) compare HHC use between enrollees in congregate living and those in other home settings. Specifically, we examined whether HHC patients in congregate living were more likely to use high-quality HHC agencies, had more planned visits, tended to be community-initiated referrals, and were more likely to be recertified for additional care episodes compared to patients in other home settings. Given disparities in access to high-quality HHC,^9^ we also assessed whether gaps in utilization among disadvantaged TM enrollees [ie, Medicare-Medicaid dually eligible individuals (duals), racial-ethnic minoritized people] and their counterparts were smaller in congregate living than in other home settings. Our results provide a deeper understanding of the interaction between HHC and congregate living.

## Methods

### Study Data and Population

We used 100% Outcome and Assessment Information Set (OASIS), which contains assessment information for all adult HHC patients in Medicare-certified agencies in 50 states and Washington, DC. We used OASIS to identify HHC episodes, agencies used, zip code, and congregate living status of each patient’s residence. These data also contain rich patient-level information (eg, health, function, availability of caregivers) and recertifications (Supplementary Appendix Data). We defined index episodes as those with a start-of-care or resumption-of-care OASIS assessment. We included all episodes that started from January 2014 to October 2019 (to allow for a sufficient follow-up period).

We linked these data with the Master Beneficiary Summary File (MBSF) to identify dual status and to restrict the sample to TM and MA enrollees who did not change insurance during their episode (60 days from beginning of care). Additionally, we used the 2018 and 2019 Chronic Condition Warehouse (CCW) file to identify chronic conditions for TM beneficiaries. Chronic condition information is not available for MA beneficiaries. To ensure adequate claims history to inform the chronic condition algorithm for TM patients, we limited our analyses to people aged 67 years and older.^13^

Finally, we used Care Compare and the Medicare Provider Utilization and Payment Data to gather agency-level information (eg, star ratings).^14,15^

### Outcome Variables: High-Quality Agencies, Planned Visits, Community-Initiated Referrals, and Recertification

Our main objectives were 2-fold. First, we aimed to describe the prevalence of HHC patients in congregate living compared with other home settings over time. Thus, we calculated the annual prevalence of TM and MA HHC episodes in congregate living from 2014 to 2019.

Our second objective was to compare the characteristics of the HHC received by Medicare patients in congregate living to other home settings. We focused on (1) use of high-quality agencies, (2) number of planned visits per episode, (3) community-initiated referral, and (4) recertification.

We defined community-initiated referrals as episodes without a hospitalization in the prior 14 days. These types of episodes have been identified by MedPAC as being a potential source of low-value HHC.^8,12^ HHC agencies were classified as high-quality if their average star rating from the Centers for Medicare & Medicaid Services exceeded 3.5 stars from 2018 to 2019; others were considered not high-quality.^9^ Prior literature suggests that high-quality agency use is associated with better patient outcomes.^16,17^ We took the planned number of visits (physical, occupational, or speech) from the OASIS. Total planned visits reflect the quantity of visits deemed to be reasonable and necessary as outlined in the treatment plan developed by the referring physician and home health therapists for each patient.^18^ As a sensitivity check, we excluded observations with planned visits above the 99th percentile (28 visits), and the results remained robust (Supplementary Table A1). Lastly, we measured whether a patient was recertified after the initial index episode (ie, whether a start-of-care or resumption-of-care episode was immediately followed by a recertification episode).

### Explanatory Variables: Congregate Living, Dual and Minoritized Status

Our explanatory variables included congregate living, dual eligibility, and race or ethnicity. We identified congregate living status (ie, residence in settings where the patient receives assistance, supervision, or oversight as part of the living arrangement) using item M1100 of OASIS (Supplementary Appendix Data).^5,18^ Examples of congregate living arrangements include apartments or rooms as part of an assisted living facility, residential care home, or personal care home.^18^ We compared TM enrollees who were duals to non-duals and minoritized people (people not non-Hispanic white) to non-Hispanic white individuals. We grouped minoritized people into a single category to improve statistical precision.

### Statistical Analysis

We used 2014–2019 data to examine trends in the prevalence of HHC patients in congregate living compared with other home settings over time. Then, restricting to 2018–2019 data, we used linear regression to quantify the association between congregate living and outcomes for HHC patients in 2018–2019, and separately for TM and MA enrollees (Supplementary Appendix Methods).

We included zip code fixed effects (binary indicators for each HHC patient’s zip code) to ensure that we only compared patients within the same zip code. This minimizes confounding from between-zip code differences, which may arise from differential access to care, neighborhood socioeconomic conditions, among other observable and unobservable factors.

Additionally, we controlled for a rich set of observable patient-level characteristics to account for population differences between residential settings. We controlled for patient demographics (age, sex, race or ethnicity), dual eligibility status, referral type (excluded in regressions examining community-initiated care), cognition, behavioral issues, health, function, and symptoms. We also controlled for the caregiver availability, as identified in OASIS, because caregivers could influence where patients reside. We used the same set of control variables for MA enrollees, except the number of CCW comorbidities, which was only available for TM enrollees. We clustered all standard errors at the zip code level. These estimates represent the average differences in outcomes between HHC patients residing in congregate living and those in other home settings, conditional on living in the same zip code and after adjustment for observable characteristics.

Finally, to assess whether outcome differences between TM patient subgroups (dual, race or ethnicity) differed between congregate living and other home settings, we estimated a modified regression model using data from 2018 to 2019 (Supplementary Appendix Methods). We stratified the TM sample into congregate living and other home settings, then analyzed the associations between outcomes and each subgroup pair. We controlled for the same aforementioned variables, excluding those that duplicated the stratifying variables. This approach enables a direct comparison of subgroup-level differences, while accounting for differences in care needs across settings and ensuring comparisons between individuals within the same zip code with similar observable demographic and health characteristics.

## Results

Among the 9,870,292 TM and MA HHC patients in 2018–2019, 1,357,470 (15.9%) resided in congregate living. Compared to other home settings, people in congregate living were less socially disadvantaged but sicker. They were more likely to be white (91.0% vs 76.8%), less likely to be dual eligible (18.0% vs 23.8), and less likely to live in rural areas (11.8% vs 21.1%). However, they had higher rates of daily cognitive and behavioral symptoms (23.2% vs 9.1%), more numbers of substantial functional limitations (5.9 vs 5.2), and more chronic conditions (6.1 vs 5.8) (Table 1).

Patients in congregate living were more likely to receive around-the-clock assistance outside of HHC (93.7% vs 70.5%), whereas those in other home settings received less frequent help. For instance, about 5% of patients in other home settings reported receiving regular daytime assistance only, compared with 1.3% of patients in congregate living (Table 1).

### Share of Medicare HHC Episodes Provided in Congregate Living, 2014 to 2019

**Fig. 1.**
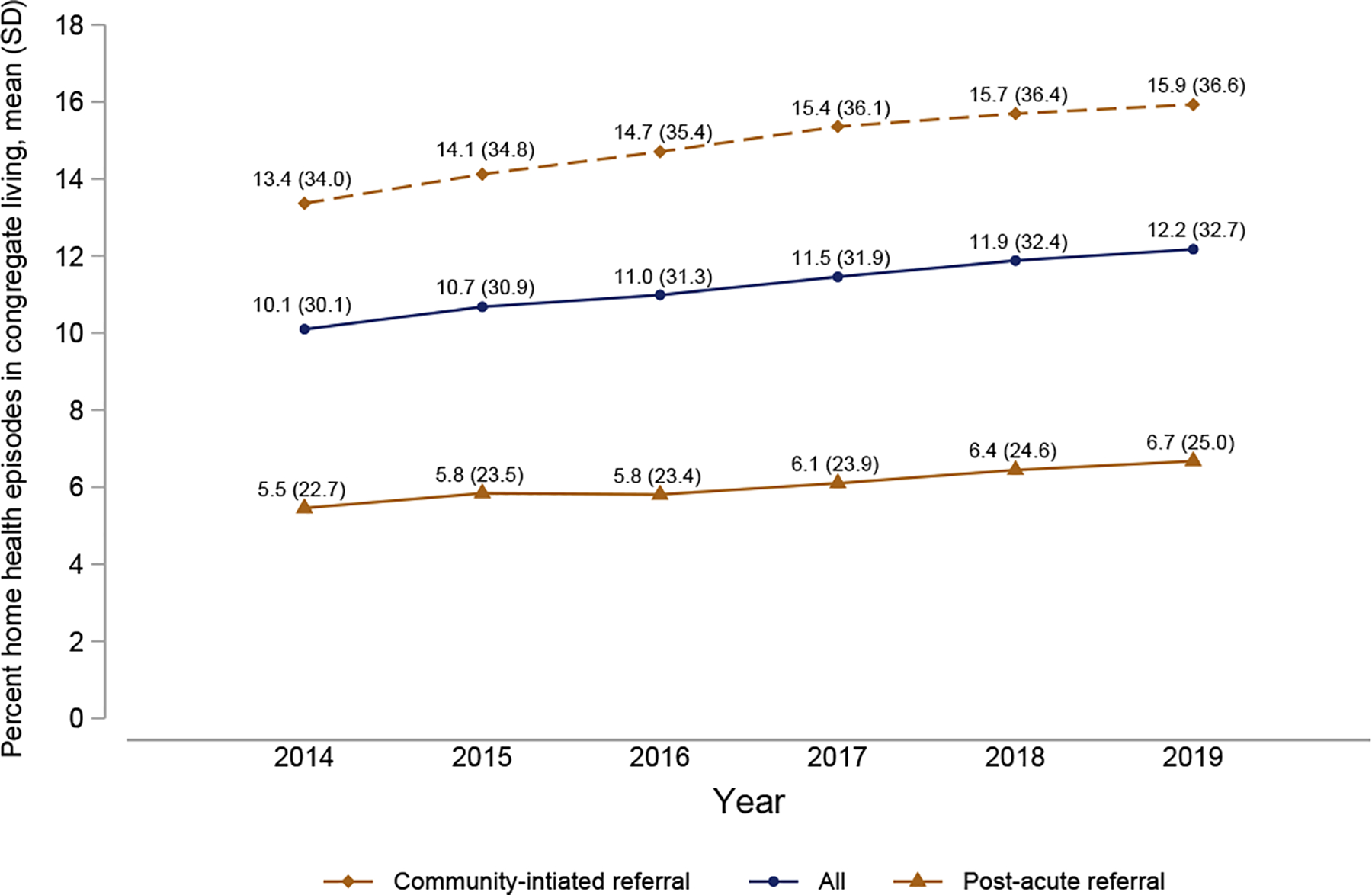
Prevalence of traditional Medicare and Medicare Advantage home health episodes in congregate living, 2014–2019. Community-initiated referrals are episodes without a hospitalization in 14 days prior to beginning of care, all else are post-acute referrals. Source: Authors’ calculations using national Medicare data, 2014–2019.

HHC patients residing in congregate living comprised an increasing share of total HHC episodes over time. In 2014, 10.1% of all episodes were provided in congregate living compared with 12.2% of episodes by 2019, a relative increase of 20.5% (Figure 1). Throughout the study window, congregate living accounted for a larger share of community-initiated episodes than post-acute episodes, although both increased with similar trajectories. By 2019, nearly 16% of all community-initiated HHC episodes occurred in congregate living, whereas 6.7% of all post-acute episodes occurred in this setting.

Notably, we find that the rate of growth in HHC use among congregate living residents far exceeded the overall growth in HHC use among all Medicare beneficiaries. Specifically, we find that total HHC episodes grew by 4.2% during the study window (from 6,704,417 to 6,987,647), whereas episodes in congregate living rose by 25.6% (from 677,276 to 850,827) (Supplementary Figure A1).

### Differences in Outcomes Between Home Health Patients in Congregate Living vs Other Home Settings

**Fig. 2.**
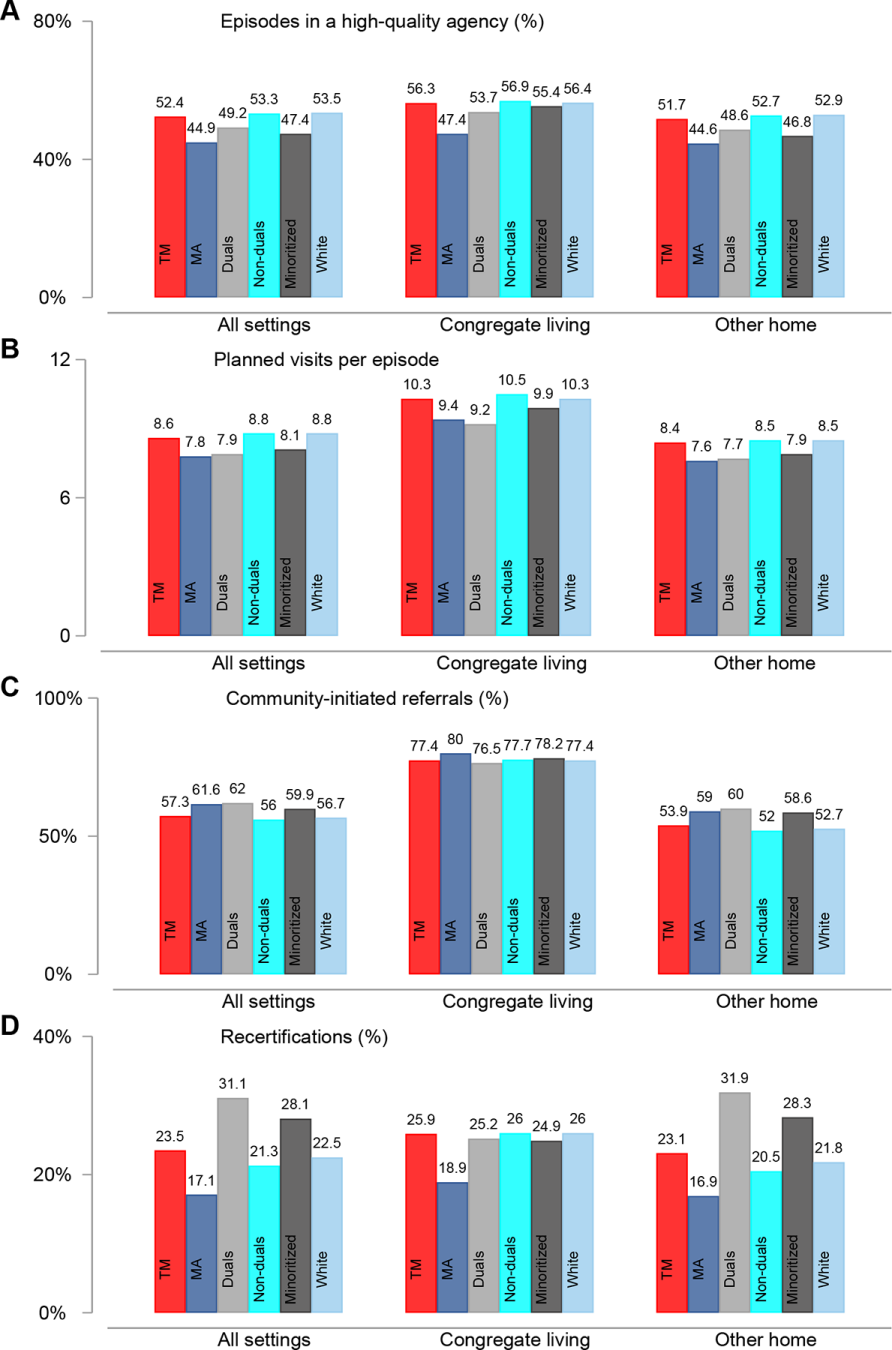
Comparison of means across Medicare subgroups in home health, 2018–2019. (A) Comparisons in terms of the percent of episodes in high-quality agencies. (B) Comparisons in terms of the average number planned visits per episode. (C) Percent of episodes that were community-initiated referrals. (D) Percent of episodes that were recertified for additional home health care. MA, Medicare Advantage; TM, traditional Medicare. Duals, non-duals, minoritized, and White people refer to subgroup of TM enrollees. Source: Authors’ calculations using national Medicare data, 2018–2019.

In unadjusted comparisons, Medicare HHC patients in congregate living were more likely to use high-quality agencies (53.4% vs 49.0%), had more planned visits per episode (10.0 vs 8.1 visits), were more likely to have community-initiated episodes (78.2% vs 55.8%), and were more often recertified (23.6% vs 20.8%) (Table 1). These patterns held for both TM and MA enrollees, although MA enrollees generally had lower averages, except for community-initiated referrals (Figure 2).

**Fig. 3.**
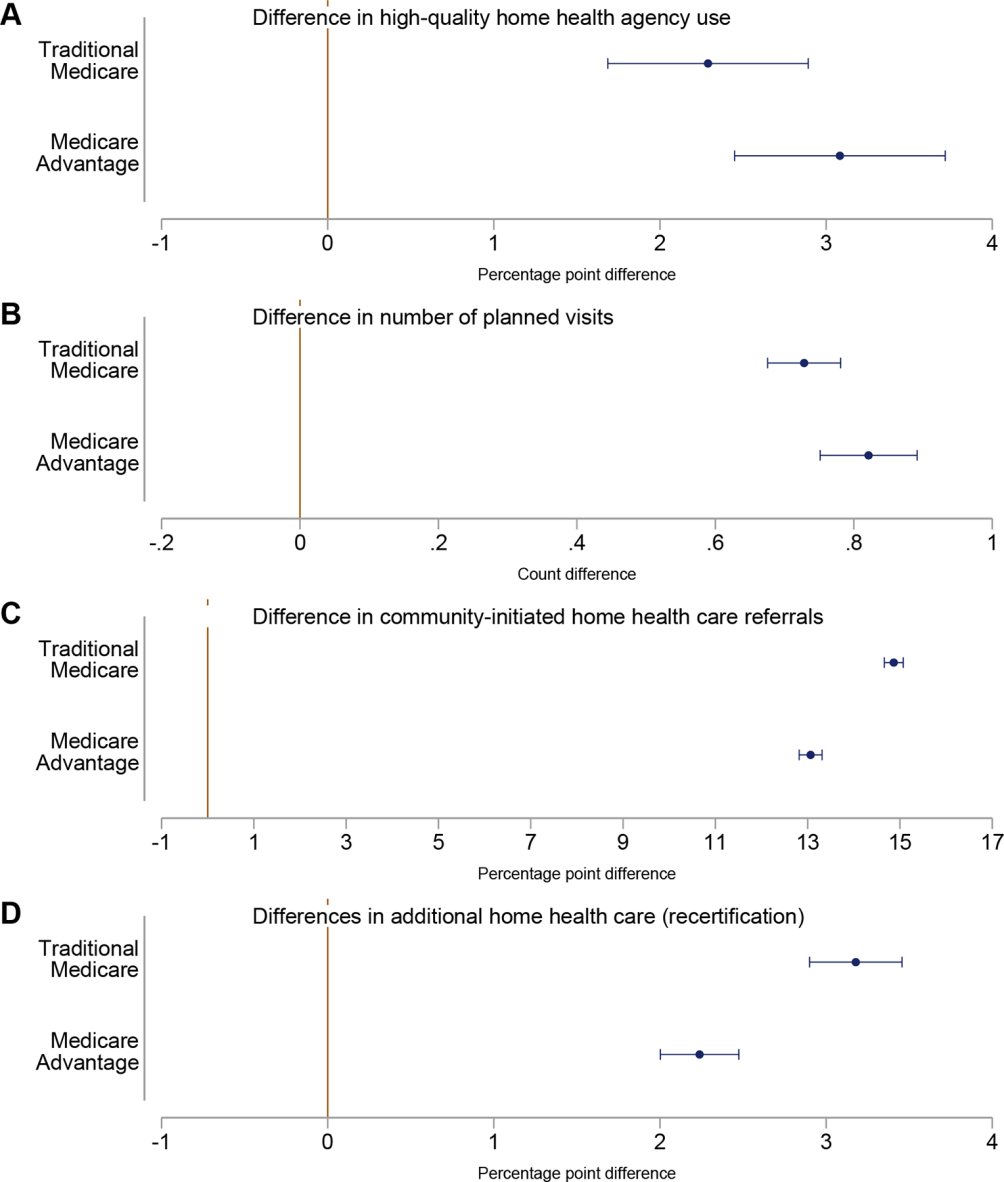
Differences in outcomes for Medicare home health patients in congregate living vs other home settings, 2018–2019. (A) Difference in likelihood of using high-quality agencies between home health care patients in congregate living vs other home settings, compared across Medicare Advantage and traditional Medicare insurance. (B) Difference in number of planned visits between home health care patients in congregate living vs other home settings, compared across Medicare Advantage and traditional Medicare insurance. (C) Difference in likelihood of community-initiated referrals between home health care patients in congregate living vs other home settings, compared across Medicare Advantage and traditional Medicare insurance. (D) Difference in likelihood of recertification for additional home health care between home health care patients in congregate living vs other home settings, compared across Medicare Advantage and traditional Medicare insurance. Comparison of differences are based on a linear regression between outcome and congregate living status for each Medicare enrollee type. Covariates include zip code fixed effects and patient characteristics on demographics, dual status, health, cognition, function, and availability of care assistance beyond those offered by home health care (Supplementary Appendix Methods). Standard errors were clustered at the zip code level. Source: Authors’ calculations using Medicare data from 2018 to 2019.

Regression-adjusted results confirmed the unadjusted differences in HHC use across settings (Figure 3) (Supplementary Table A2). Compared with those in other home settings, TM patients in congregate living were 4.4% (2.3 ppt, 95% CI: 1.7, 2.9) more likely to use a high-quality agency, had 8.7% (0.7 visits, 95% CI: 0.7, 0.8) more planned visits, were 27.6% (14.9 ppt, 95% CI: 14.7, 15.1) more likely to have community-initiated episodes, and 9.9% (3.2 ppt, 95% CI: 2.9, 3.5) more often recertified.

Differences in outcomes between settings among MA enrollees were also statistically significant and directionally similar to those among TM enrollees (Figure 3) (Supplementary Table A2). On community-initiated referrals and recertifications, differences between settings for MA enrollees were statistically smaller than for TM enrollees.

### Differences in Outcomes Between Subgroups of TM Patients

**Fig. 4.**
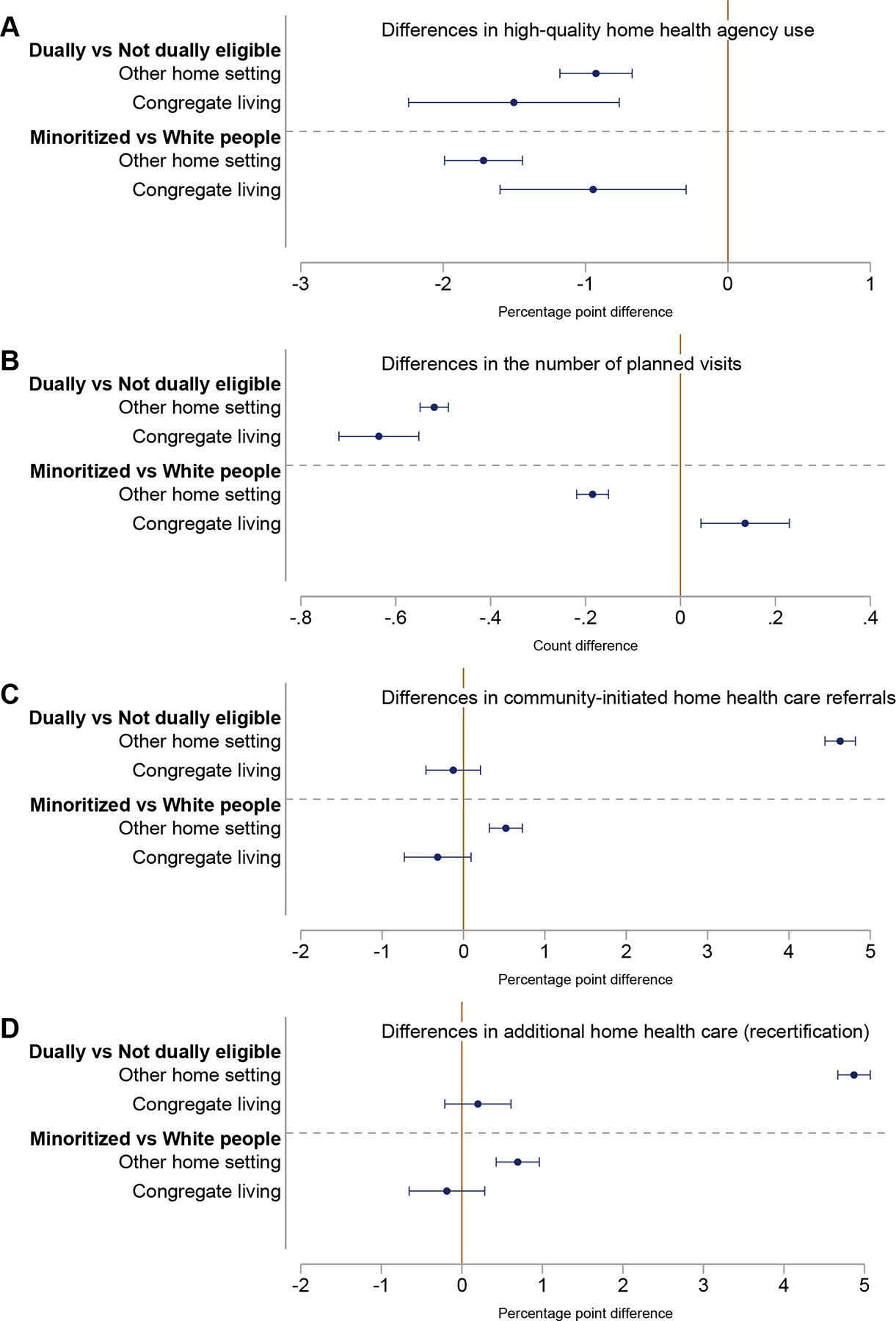
Differences in outcomes between subgroups of traditional Medicare home health patients in congregate living and other home settings, 2018–2019. (A) Difference in likelihood of using high-quality agencies among traditional Medicare home health care patients, comparing (1) dually eligible vs not dually eligible patients and (2) minoritized vs White patients, both stratified by congregate living status. (B) Difference in number of planned visits among traditional Medicare home health care patients, comparing (1) dually eligible vs not dually eligible patients and (2) minoritized vs White patients, both stratified by congregate living status. (C) Difference in likelihood of community-initiated referrals among traditional Medicare home health care patients, comparing (1) dually eligible vs not dually eligible patients and (2) minoritized vs White patients, both stratified by congregate living status. (D) Difference in likelihood of recertification for additional home health care among traditional Medicare home health care patients, comparing (1) dually eligible vs not dually eligible patients and (2) minoritized vs White patients, both stratified by congregate living status. Comparison of differences are based on a linear regression between outcome and subgroup (ie, dual vs not dual, minoritized vs White people) for each living setting. Covariates include zip code fixed effects and patient characteristics including demographics, health, cognition, function, and availability of care assistance beyond those offered by home health care (see Supplementary Appendix Methods) . Standard errors were clustered at the zip code level. Source: Authors’ calculations using Medicare data from 2018 to 2019.

Duals and minoritized people generally used HHC differently from their counterparts in both settings. For instance, combining both settings, the use of high-quality agencies was 4.1 ppt (8.3%) lower among duals than non-duals and 6.1 ppt (12.9%) lower among minoritized than white people (Figure 2). After controlling for a variety of patient characteristics, duals and minoritized people were still less likely to use high-quality agency in both settings. Among people in congregate living, for instance, duals were 2.6% less likely (−1.5 ppt, 95% CI: −2.2, −0.8%), and minoritized people were 1.6% less likely (−0.9 ppt, 95% CI: −1.6, −0.3%) to use high-quality agencies than their counterparts (Figure 4, Supplementary Tables A3 and A4).

The number of planned visits was also lower, by about 6%, for duals relative to non-duals, regardless of setting. Duals received 0.64 fewer planned visits (95% CI: −0.72, −0.55) in congregate living and 0.52 fewer planned visits (95% CI: −0.55, −0.49) in other home settings (Figure 4, Supplementary Table A3). Differences in the number of planned visits between minoritized and white people were much smaller, albeit statistically significant, in both settings (1.3% increase in congregate living, 2.2% decrease in other home settings) (Figure 4, Supplementary Table A4).

In contrast, duals and minoritized people in other home settings had higher rates for community-initiated episodes and recertification. Compared with non-duals, duals had 8.9% (4.6 ppt, 95% CI: 4.4, 4.8 ppt) higher rates of community-initiated referrals and 23.9% (4.9 ppt, 95% CI: 4.7, 5.1 ppt) higher recertification rates (Figure 4, Supplementary Table A3). Although differences were modest, relative to white people, minoritized people had 0.9% (0.5 ppt, 95% CI: 0.3, 0.7 ppt) higher rates of community-initiated referrals and 3.2% (0.7 ppt, 95% CI: 0.4, 1.0 ppt) higher recertification rates (Figure 4, Supplementary Table A4). In congregate living, however, no group-level differences were found in community-initiated episodes or recertification rates. For all groups in congregate living, the rates were approximately 75% and 25%, respectively (Figure 2).

The observed patterns in group differences in community-initiated episodes and recertificationsdsmall gaps in congregate living and large gaps in other home settingsdwere primarily due to higher rates among non-duals and white people in congregate living. For instance, the recertification rate for non-duals in congregate living (26.0%) was 26.8% higher than in other home settings (20.5%), aligning with the rate among duals (25.2%) in congregate living. In contrast, the recertification rate for duals was not higher in congregate living and was 21% lower compared with other home settings (31.9%) (Figure 2).

## Discussion

From 2014 to 2019, the number of Medicare HHC episodes in congregate living increased by nearly 26%. At the end of our study window, more than 12% of all Medicare HHC episodes were delivered in this setting. For both TM and MA HHC patients, congregate living was associated with a greater likelihood of using high-quality agencies, more planned visits, more community-initiated referrals, and higher recertification rates. However, regardless of setting, duals and minoritized people were slightly less likely (about 2%) to use high-quality agencies, and duals had about 6% fewer planned visits than non-duals. Additionally, duals, and, to a lesser extent, minoritized people, had higher rates of community-initiated referrals and recertifications than their counterparts, but only in other home settings. In congregate living, there were no detectable differences.

Our results highlight the growing importance of the co-use of congregate living and HHC. According to the National Study of Long-Term Care Providers, the number of congregate living residents increased by 10% from 835,200 in 2014 to 918,700 in 2018.^19,20^ However, the growth in HHC episodes within congregate living, as documented in this study, has considerably outpaced this population growth. These trends may reflect the complementary nature of both services, each of which serve individuals who cannot live independently but do not require continuous institutional care.

Less clear from our data is the clinical value to patients of extensive HHC in congregate living. On one hand, simultaneous use of both types of services may benefit patients, especially if congregate living providers assist with identifying the need for HHC and connecting patients with high-quality providers. Our findings suggest that congregate living residents were more likely to receive care from high-quality agencies and typically received more care—both in terms of planned visits and recertifications. These patterns persisted after controlling for a rich set of patient characteristics, suggesting that these associations may not solely be due to congregate living residents being sicker or more disabled. Instead, this may speak to the role of congregate living providers as intermediaries who help improve access to high-quality HHC.

On the other hand, congregate living was linked to substantially higher rates of community-initiated episodes and recertifications, particularly among non-duals and white people, for whom these rates were relatively low in other home settings but high in congregate living and on par with rates among duals and minoritized people. MedPAC has identified community-initiated episodes and recertifications as care that is at risk of being inappropriately used as a long-term care benefit and has previously suggested imposing a patient copays on these episodes to discourage unnecessary use.^8,12^ Ascertaining the clinical appropriateness of these episodes is beyond the scope of this article but is an important area for future study.

Regardless of the role of congregate living, our study shows that socially disadvantaged patients, especially duals, appear worse off than their counterparts. For instance, duals were less likely to use high-quality HHC agencies and had fewer planned visits per episode, regardless of setting. Even on community-initiated referrals and recertifications, where we observed no gaps between groups in congregate living, the parity was largely due to non-duals “catching up” to duals. Rather than reflecting greater access to HHC, the consistently high rates of community-initiated referrals and recertifications among duals and minoritized people may instead be partially capturing barriers to long-term care alternatives. This is particularly troubling since HHC is only provided intermittently (no more than 8 hours per day) and may be insufficient for people with more substantial needs.^21–23^

This study has limitations. These estimates are descriptive and not causal. Although we controlled for a rich set of patient-level characteristics and zip code fixed effects, there are likely residual differences between groups (ie, residents in congregate living vs other home settings, duals vs non-duals, and minoritized vs white people) that contribute to the observed outcome differences. Additionally, although we examined many outcomes, we were unable to disentangle mechanisms. Moreover, we were unable to examine actual visits rendered. Although planned visits capture the quantity of visits deemed necessary and appropriate by the physician and therapist in the patient care plan, it presents an incomplete picture of care delivery. Lastly, congregate living status is documented by each agency’s clinicians and may contain error despite detailed documentation and auditing procedures. These limitations notwithstanding, our study provides important descriptive information to guide future studies for understanding how these two sectors can be leveraged to serve patients.

## Conclusions and Implications

Our findings suggest that the combination of congregate living and HHC is becoming more common in the United States. Although we found a correlation between congregate living and access to high-quality HHC, results may be consistent with some overuse of HHC as well as possible inequitable distribution of benefits. As the aging population relies more on community-based services, understanding the interactions between service sectors will be increasingly important in the coming years.

## Supplementary Material

Appendix

## Figures and Tables

**Table 1:** Characteristics of Medicare Home Health Patients in Congregate Living vs Other Home Settings, 2018–2019

	Congregate Living, Mean (SD)	Other Home Settings, Mean (SD)
Outcomes		
High-quality agencies	0.534 (0.499)	0.490 (0.500)
Planned visits (count)	10.0 (8.9)	8.1 (8.7)
Community-initiated referrals	0.782 (0.413)	0.558 (0.497)
Recertification	0.236 (0.425)	0.208 (0.406)
Patients		
Demographics		
Age (y)	85.5 (7.9)	80.1 (8.0)
Female	0.686 (0.464)	0.612 (0.487)
Hispanic	0.033 (0.180)	0.076 (0.266)
White	0.910 (0.286)	0.768 (0.422)
Black	0.040 (0.196)	0.124 (0.330)
Asian	0.014 (0.116)	0.026 (0.159)
Other	0.003 (0.058)	0.006 (0.076)
Dually eligible	0.180 (0.384)	0.238 (0.426)
Health		
CCs (count)	6.1 (4.4)	5.8 (4.6)
Constant pain	0.122 (0.327)	0.183 (0.387)
No cognitive/behavioral symptoms	0.414 (0.492)	0.725 (0.447)
Daily cognitive/behavioral symptoms	0.232 (0.422)	0.091 (0.287)
ADL impairment (count)	8.6 (1.0)	8.4 (1.3)
Substantial ADL impairment count (count)	5.9 (2.2)	5.2 (2.4)
Home environment		
Rural	0.118 (0.323)	0.211 (0.408)
Around the clock assistance	0.937 (0.243)	0.705 (0.456)
Regular daytime assistance	0.013 (0.113)	0.050 (0.219)
Regular nighttime assistance	0.001 (0.033)	0.054 (0.226)
Short-term assistance	0.047 (0.213)	0.182 (0.386)
No available assistance	0.002 (0.041)	0.009 (0.094)
Home health agency		
Patients (count)	1490 (1948)	1938 (3125)
Episodes (count)	2354 (2883)	2911 (4411)
For-profit	0.759 (0.428)	0.600 (0.490)
Operational duration (year)	23.0 (14.1)	27.8 (14.9)
Dually eligible (%)	20.7 (15.3)	22.7 (16.4)
Dually eligible (%)	20.7 (15.3)	22.7 (16.4)
Patient age (year)	78.4 (3.2)	76.7 (2.6)
Female (%)	61.5 (4.9)	60.2 (4.2)
Hispanic (%)	7.4 (14.4)	9.0 (16.4)
White (%)	82.0 (15.8)	77.6 (18.6)
Black (%)	9.6 (10.2)	13.1 (13.6)
Asian (%)	3.4 (8.2)	3.9 (10.0)
Other (%)	1.1 (1.8)	1.6 (3.2)
Patient risk score (count)	2.3 (0.3)	2.3 (0.3)
CCs (count)	5.6 (0.6)	5.6 (0.6)
Rural patients (%)	16.7 (24.3)	22.7 (29.8)

ADL, activities of daily living; CCs, chronic conditions.

All estimates show rates unless otherwise specified. Sample included 9,870,292 total episodes, with 1,357,470 in congregate living and 8,512,822 in other home settings.
