# Supplementary material for: Trends in the Use of Medicare Home Health Care among Congregate Living Residents
Source: J Am Med Dir Assoc. Author manuscript; Available in PMC 2025 Apr 7. (PMC11972877; doi:10.1016/j.jamda.2025.105498)
Supplement: Appendix [file NIHMS2063289-supplement-Appendix.pdf]

Appendices

Data. OASIS items used in study P.2
Methods. Estimating equations P.5
Tables A1-A4 P.7
Figure A1 P.11

Appendix Data. OASIS items used in study

OASIS Item	Description	Use
M0100 This Assessment is Currently Being Completed for the Following Reason:<br>[1] Start of care – further visits planned<br>[3] Resumption of care (after inpatient stay)<br>[4] Recertification (follow-up) reassessment	Identify beginning of home health episode	Sample, Outcome
M2200 Therapy Need	Number of planned visits. Therapy visits must (a) relate directly and specifically to a treatment regimen established by the physician through consultation with the therapist(s), and (b) be reasonable and necessary to the treatment of the patient’s illness or injury	Outcome
M1100 Patient Living Situation: Which of the following best describes the patient's residential circumstance and availability of assistance?<br>[a] Patient lives alone<br>[b] Patient lives with other person(s) in the home<br>[c] Patient lives in congregate situation (for example: assisted living, residential care home).	Congregate living or other home setting. According to CMS, patients are in a congregate living situation if “the patient lives in an ‘assisted living’ setting (assistance, supervision and/or oversight are provided as part of the living arrangement).” It has also been used in prior literature to identify home health care patients in assisted living.	Predictor
M0140 Race/Ethnicity (Mark all that apply)<br>[1] American Indian or Alaska Native<br>[2] Asian<br>[3] Black or African-American<br>[4] Hispanic or Latino<br>[5] Native Hawaiian or Pacific Islander<br>[6] White	Minoritized people (people who are not non-Hispanic White) or White people (non-Hispanic White)	Predictor, covariate
M0060 Patient ZIP Code	Identify patient ZIP code	Covariate
M0069 Gender	Female/male	Covariate
M1100 Patient Living Situation (Availability of Assistance)<br>[01, 06, 11] Around the clock<br>[02, 07, 12] Regular daytime<br>[03, 08, 13] Regular nighttime<br>[04, 09, 14] Occasional/short-term assistance<br>[05, 10, 15] No assistance available	Determine availability of help outside of those provided by the home health agency	Covariate
M1242 Pain Frequency<br>[0] No pain<br>[1] Pain does not interfere<br>[2] Less often than daily<br>[3] Daily, but not constantly	Symptoms/Health	

[4] All of the time		
M1306 Unhealed Pressure Ulcer at Stage 2 or Higher	Symptoms/Health	Covariate
M1322 Number of Stage 1 Pressure Ulcers	Symptoms/Health	Covariate
M1400 When is the patient dyspneic	Symptoms/Health	Covariate
M1610 Urinary Incontinence or Urinary Catheter Presence	Symptoms/Health	Covariate
M1620 Bowel Incontinence Frequency	Symptoms/Health	Covariate
M1700 Current Day Cognitive Functioning<br>[0] Alert<br>[1] Requires prompting<br>[2] Requires assistance and direction<br>[3] Requires considerable assistance<br>[4]Total dependent	Cognitive impairment	Covariate
M1710 When Confused in last 14 Days<br>[0] Never<br>[1] In new/complex situations only<br>[2] On awaking or night<br>[3] During day and evening but not constantly<br>[4] Constantly<br>[NA] Nonresponsive	Cognitive impairment	Covariate
M1720 When Anxious in Last 14 Days<br>[0] Never<br>[1] Less often than daily<br>[2] Daily, but not constantly<br>[3] All the time<br>[4] Nonresponsive	Cognitive impairment	Covariate
M1740 Cognitive, behavioral, psychiatric symptoms demonstrated at least weekly<br>[1] Memory deficit<br>[2] Impaired decision-making<br>[3] Verbal disruption<br>[4] Physical aggression<br>[5] Socially inappropriate behavior<br>[6] Delusional<br>[7] None	Cognitive impairment	Covariate
M1745 Frequency of Disruptive Behavior Symptoms<br>[0] Never<br>[1] Less than once a month<br>[2] Once a month<br>[3] Several times each month<br>[4] Several times a week<br>[5] At least daily	Cognitive impairment	Covariate
M1800 Grooming<br>[0] Independent<br>[1] Grooming utensils must be placed within reach<br>[2] Someone must assist the patient	Stages of limitation<br>Any: [>0]<br>Severe limitation: [>=2]	Covariate

[3] Patient depends entirely upon someone else		
M1810 Dress Upper Body<br>[0] Able without assistance<br>[1] Without assistance if clothes laid out<br>[2] Someone must help<br>[3] Depends entirely on others	Stages of limitation<br>Any limitation: [>0]<br>Severe limitation: [>=2]	Covariate
M1820 Dress Lower Body<br>[0] Independent<br>[1] Without assistance if clothes laid out<br>[2] Someone must help<br>[3] Depends entirely on others	Stages of limitation<br>Any limitation: [>0]<br>Severe limitation: [>=2]	Covariate
M1830 Bathing<br>[0] Able independently<br>[1] With use of devices<br>[2] Intermittent assistance<br>[3] Requires continued presence<br>[4] Independent only in select settings<br>[5] Need assistance in select settings<br>[6] Depends entirely on others	Stages of limitation<br>Any limitation: [>0]<br>Severe limitation: [>=3]	Covariate
M1840 Toilet Transferring<br>[0] Independent<br>[1] Assistance needed<br>[2] Bedside commode<br>[3] Bedside commode with assistance<br>[4] Depends entirely on others	Stages of limitation<br>Any limitation: [>0]<br>Severe limitation: [>=2]	Covariate
M1845 Toilet Hygiene<br>[0] Independent<br>[1] Without assistance if supplies laid out<br>[2] Someone must help<br>[3] Depends entirely on others	Stages of limitation<br>Any limitation: [>0]<br>Severe limitation: [>=2]	Covariate
M1850 Bed Transferring<br>[0] Able independently<br>[1] With use of devices<br>[2] Can bear weight but not transfer<br>[3] Unable to bear weight<br>[4] Bedfast, can turn<br>[5] Bedfast, unable to turn	Stages of limitation<br>Any limitation: [>0]<br>Severe limitation: [>=2]	Covariate
M1860 Ambulation<br>[0] Able independently<br>[1] With use of one-handed devices<br>[2] With use of two-handed devices<br>[3] Requires continued presence<br>[4] Can wheel independently<br>[5] Cannot wheel self<br>[6] Bedfast, unable to ambulate	Stages of limitation<br>Any limitation: [>0]<br>Severe limitation: [>=3]	Covariate

M1870 Feeding<br>[0] Independent<br>[1] Independent but requires set up or assistance<br>[2] Unable to feed<br>[3]Able to feed orally and require tube feeding<br>[4] Unable to feed orally and require tube feeding<br>[5] Unable to feed orally or by tube	Stages of limitation<br>Any limitation: [>0]<br>Severe limitation: [>=2]	Covariate

Appendix Methods. Estimating equations
We estimated equation (1), separately by enrollees of each payer, $p \in \{TM, MA\}$

$$y_{iz} = \alpha_1^p Congregate_i + \boldsymbol{X\beta^p} + \zeta_z^p + \epsilon_{iz} \quad (1)$$

where y_{iz} is the outcome for Medicare home health episode i for the patient in ZIP code z in 2018 and 2019. $Congregate_i$ is a binary indicator variable equal to 1 if the patient was in congregate living and 0 in other home settings. $\boldsymbol{X\beta^p}$ represents a vector of baseline patient characteristics, including:

- Demographics: Age, age squared, race/ethnicity, sex
- Medicare-Medicaid dual-eligibility status
- Health: Number of CCW comorbidities, dementia diagnosis, community-initiated or post-acute referral type (for all outcomes except for community-initiated care)
- Symptoms: Pressure ulcers, shortness of breath, urinary and bowel incontinence, pain
- Functional limitations: Ambulation, grooming, dressing, bathing, toileting, eating
- Cognitive issues: cognitive functioning, confusion, anxiety, delusions, impaired decision making, memory deficits, physical aggression, socially inappropriate behavior, verbal aggression
- Availability of in-person caregivers

This vector of covariates excluded the number of CCW comorbidities and dementia diagnosis for Medicare Advantage enrollees.
ζ_z is a binary indicator variable for each ZIP code in the sample. All standard errors were clustered at the ZIP code level.
In this equation, $\hat{\alpha}_1^p > 0$ suggests a higher value in outcome y_{iz} between home health patients of payer p in congregate living versus other home settings.

We estimated equation (2), separately for TM enrollees by living setting, $c \in \{Congregate\ living, other\ home\ settings\}$

$$y_{iz} = \delta_1^c Subgroup_{iz} + \boldsymbol{X\beta^c} + \zeta_z^c + \nu_{iz} \quad (2)$$

where y_{iz} is the outcome for the TM patient in home health episode i residing in ZIP code z in 2018 and 2019. $Subgroup$ is a binary indicator variable equal to 1 if the patient was in a given subgroup pair and 0 otherwise, including dual vs. non-dual, minoritized vs. White people, and PLWD vs without dementia. $\boldsymbol{X\beta^c}$ represents a similar vector of baseline characteristics as equation (1), excluding the variables (dual status, race/ethnicity) that overlapped with a given subgroup pair.
In this equation, $\hat{\delta}_1^c > 0$ suggests a higher value in outcome y_{iz} for a given subgroup (i.e., dual, minoritized people) compared to its counterpart (i.e., non-dual, White people) within setting c.

Appendix Tables

Table A1. Estimated differences across samples using truncated planned therapy visits

Outcome	Coef.(SE)	95% CI	P-value
CL vs other (TM)	0.686(0.024)	(0.639, 0.732)	(0.000)
CL vs other (MA)	0.733(0.027)	(0.680, 0.787)	(0.000)
Dual vs non-dual (CL)	-0.537(0.037)	(-0.610, -0.464)	(0.000)
Dual vs non-dual (other)	-0.483(0.013)	(-0.508, -0.457)	(0.000)
Minoritized vs White (CL)	0.146(0.043)	(0.062, 0.229)	(0.001)
Minoritized vs White (other)	-0.173(0.015)	(-0.202, -0.143)	(0.000)

Notes: CL – Congregate living, other – other home settings, TM – Traditional Medicare, MA – Medicare Advantage. Covariates include full set of demographic, health, function, cognition, symptoms, availability of caregivers, and ZIP code fixed effects. Standard errors clustered at the ZIP code level. Each row is a single regression

Table A2. Regression adjusted estimates across various statistical models examining differences in outcomes between Medicare home health patients in congregate living versus other home settings.

Outcome	Covariates	Coef.(SE)	95% CI	P-value
Traditional Medicare				
High-quality agency	None	0.025(0.003)	(0.019, 0.032)	(0.000)
High-quality agency	Basic	0.023(0.003)	(0.017, 0.029)	(0.000)
High-quality agency	All	0.023(0.003)	(0.017, 0.029)	(0.000)
Planned visits	None	1.520(0.029)	(1.463, 1.578)	(0.000)
Planned visits	Basic	0.918(0.028)	(0.864, 0.972)	(0.000)
Planned visits	All	0.728(0.027)	(0.676, 0.781)	(0.000)
Community-initiated referral	None	0.220(0.001)	(0.217, 0.222)	(0.000)
Community-initiated referral	Basic	0.160(0.001)	(0.158, 0.162)	(0.000)
Community-initiated referral	All	0.149(0.001)	(0.147, 0.151)	(0.000)
Recertification	None	0.057(0.001)	(0.054, 0.060)	(0.000)
Recertification	Basic	0.041(0.001)	(0.038, 0.044)	(0.000)
Recertification	All	0.032(0.001)	(0.029, 0.035)	(0.000)
Medicare Advantage				
High-quality agency	None	0.040(0.004)	(0.033, 0.047)	(0.000)
High-quality agency	Basic	0.036(0.003)	(0.029, 0.043)	(0.000)
High-quality agency	All	0.031(0.003)	(0.024, 0.037)	(0.000)
Planned visits	None	1.461(0.040)	(1.382, 1.541)	(0.000)
Planned visits	Basic	1.104(0.038)	(1.028, 1.179)	(0.000)
Planned visits	All	0.821(0.036)	(0.751, 0.891)	(0.000)
Community-initiated referral	None	0.198(0.001)	(0.195, 0.201)	(0.000)
Community-initiated referral	Basic	0.158(0.001)	(0.155, 0.161)	(0.000)
Community-initiated referral	All	0.131(0.001)	(0.128, 0.133)	(0.000)
Recertification	None	0.036(0.001)	(0.034, 0.039)	(0.000)
Recertification	Basic	0.032(0.001)	(0.030, 0.034)	(0.000)
Recertification	All	0.022(0.001)	(0.020, 0.025)	(0.000)

Notes: TM: 6,236,742 episodes in 143,873 ZIP codes; MA: 3,633,665 episodes in 107,696 ZIP codes. None-No covariates except for ZIP code fixed effects. Basic-Also includes age, age squared, sex, race/ethnicity, dual eligibility status, referral type, dementia (not included for MA models), number of comorbidities (not included for MA models). All-Also includes full set of health, function, cognition, symptoms, availability of caregivers. All outcomes represent rates except for planned visits (counts). All standard errors clustered at the ZIP code level. Each row is a single regression

Table A3. Regression adjusted estimates across various statistical models examining differences in outcomes between TM home health patients who were dually eligible versus not, 2018-2019.

Outcome	Covariates	Coef.(SE)	95% CI	P-value
Congregate living				
High-quality agency	None	-0.017(0.004)	(-0.025, -0.010)	(0.000)
High-quality agency	Basic	-0.017(0.004)	(-0.025, -0.009)	(0.000)
High-quality agency	All	-0.015(0.004)	(-0.022, -0.008)	(0.000)
Planned visits	None	-0.736(0.046)	(-0.826, -0.645)	(0.000)
Planned visits	Basic	-0.701(0.045)	(-0.789, -0.613)	(0.000)
Planned visits	All	-0.635(0.043)	(-0.720, -0.551)	(0.000)
Community-initiated referral	None	-0.017(0.002)	(-0.020, -0.013)	(0.000)
Community-initiated referral	Basic	-0.001(0.002)	(-0.005, 0.002)	(0.481)
Community-initiated referral	All	-0.001(0.002)	(-0.005, 0.002)	(0.461)
Recertification	None	0.009(0.002)	(0.005, 0.013)	(0.000)
Recertification	Basic	0.004(0.002)	(-0.000, 0.008)	(0.055)
Recertification	All	0.002(0.002)	(-0.002, 0.006)	(0.341)
Other home settings				
High-quality agency	None	-0.011(0.001)	(-0.014, -0.009)	(0.000)
High-quality agency	Basic	-0.007(0.001)	(-0.010, -0.005)	(0.000)
High-quality agency	All	-0.009(0.001)	(-0.012, -0.007)	(0.000)
Planned visits	None	-0.479(0.016)	(-0.512, -0.447)	(0.000)
Planned visits	Basic	-0.559(0.016)	(-0.590, -0.528)	(0.000)
Planned visits	All	-0.519(0.015)	(-0.549, -0.489)	(0.000)
Community-initiated referral	None	0.044(0.001)	(0.042, 0.046)	(0.000)
Community-initiated referral	Basic	0.061(0.001)	(0.059, 0.063)	(0.000)
Community-initiated referral	All	0.046(0.001)	(0.044, 0.048)	(0.000)
Recertification	None	0.066(0.001)	(0.064, 0.068)	(0.000)
Recertification	Basic	0.063(0.001)	(0.060, 0.065)	(0.000)
Recertification	All	0.049(0.001)	(0.047, 0.051)	(0.000)

Notes: Congregate living: 914,176 episodes in 20,445 ZIP codes; Other home settings: 5,312,551 episodes in 136,718 ZIP codes. None-No covariates except for ZIP code fixed effects. Basic-Also includes age, age squared, sex, race/ethnicity, referral type, dementia, number of comorbidities. All-Also includes full set of health, function, cognition, symptoms, availability of caregivers. All outcomes represent rates except for planned visits (counts). All standard errors clustered at the ZIP code level. Each row is a single regression

Table A4. Regression adjusted estimates across various statistical models examining differences in outcomes between TM home health patients who were minoritized versus White people, 2018-2019.

Outcome	Covariates	Coef.(SE)	95% CI	P-value
Congregate living				
High-quality agency	None	-0.014(0.004)	(-0.021, -0.007)	(0.000)
High-quality agency	Basic	-0.012(0.003)	(-0.018, -0.005)	(0.001)
High-quality agency	All	-0.009(0.003)	(-0.016, -0.003)	(0.005)
Planned visits	None	0.021(0.052)	(-0.081, 0.123)	(0.684)
Planned visits	Basic	0.150(0.051)	(0.051, 0.249)	(0.003)
Planned visits	All	0.136(0.048)	(0.043, 0.229)	(0.004)
Community-initiated referral	None	-0.001(0.002)	(-0.006, 0.003)	(0.580)
Community-initiated referral	Basic	-0.001(0.002)	(-0.005, 0.004)	(0.789)
Community-initiated referral	All	-0.003(0.002)	(-0.007, 0.001)	(0.129)
Recertification	None	-0.004(0.003)	(-0.009, 0.001)	(0.113)
Recertification	Basic	-0.005(0.002)	(-0.010, -0.000)	(0.035)
Recertification	All	-0.002(0.002)	(-0.007, 0.003)	(0.439)
Other home settings				
High-quality agency	None	-0.022(0.001)	(-0.025, -0.019)	(0.000)
High-quality agency	Basic	-0.020(0.001)	(-0.023, -0.017)	(0.000)
High-quality agency	All	-0.017(0.001)	(-0.020, -0.014)	(0.000)
Planned visits	None	-0.294(0.018)	(-0.330, -0.258)	(0.000)
Planned visits	Basic	-0.156(0.018)	(-0.191, -0.120)	(0.000)
Planned visits	All	-0.185(0.017)	(-0.219, -0.152)	(0.000)
Community-initiated referral	None	0.019(0.001)	(0.017, 0.021)	(0.000)
Community-initiated referral	Basic	0.005(0.001)	(0.003, 0.007)	(0.000)
Community-initiated referral	All	0.005(0.001)	(0.003, 0.007)	(0.000)
Recertification	None	0.027(0.001)	(0.024, 0.029)	(0.000)
Recertification	Basic	0.009(0.001)	(0.006, 0.011)	(0.000)
Recertification	All	0.007(0.001)	(0.004, 0.010)	(0.000)

Notes: Congregate living: 914,176 episodes in 20,445 ZIP codes; Other home settings: 5,312,551 episodes in 136,718 ZIP codes. None-No covariates except for ZIP code fixed effects. Basic-Also includes age, age squared, sex, dual eligibility status, referral type (not included for community-initiated referral models), dementia, number of comorbidities. All-Also includes full set of health, function, cognition, symptoms, availability of caregivers. All outcomes represent rates except for planned visits (counts). All standard errors clustered at the ZIP code level. Each row is a single regression

Appendix Figure

Figure A1. Total TM or MA home health episodes by home health referral type, 2014-2019

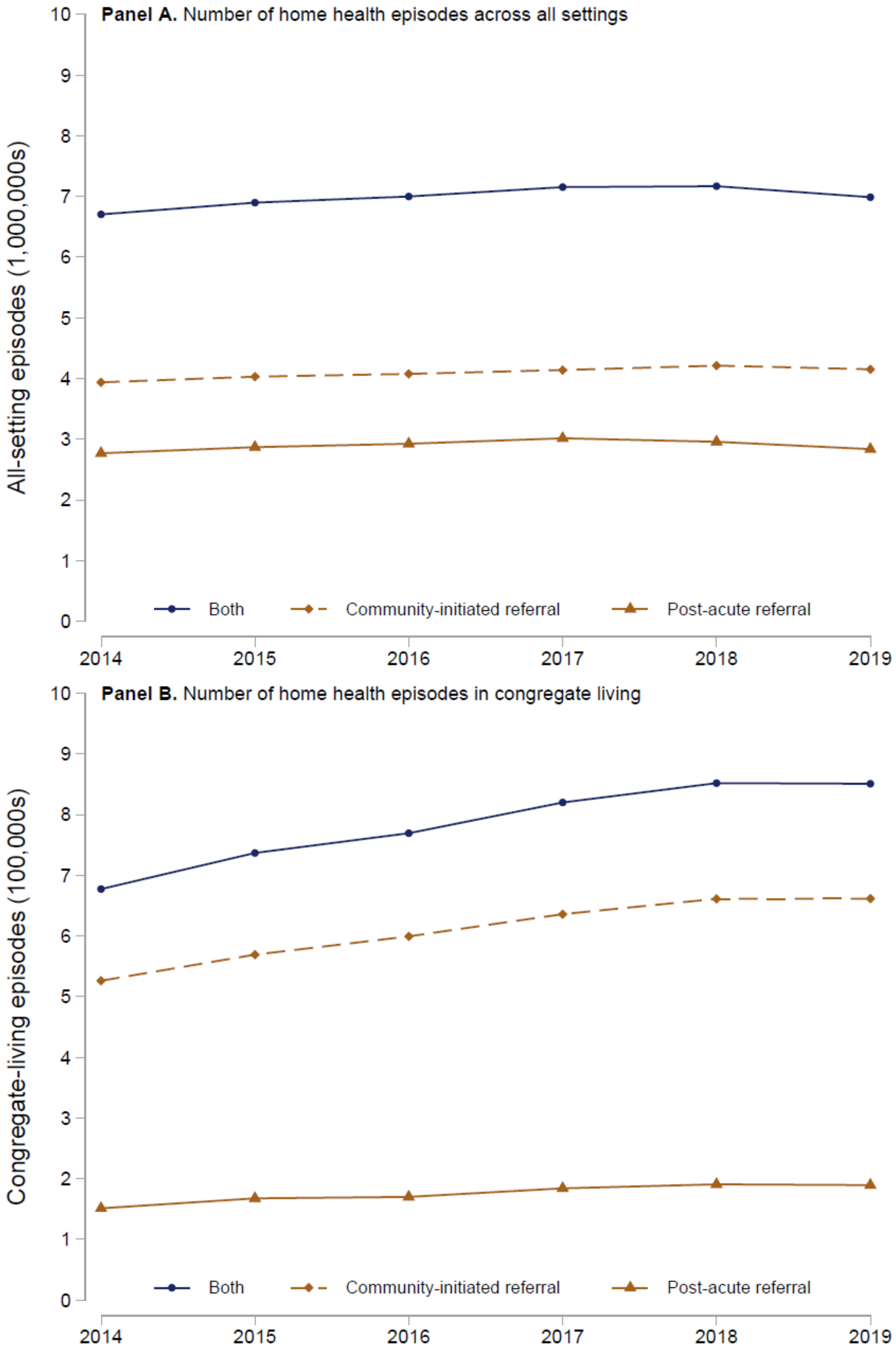

Notes: Community is community-initiated referral to home health.
